# Nodular pulmonary amyloidosis presenting as a localized manifestation of lymphoproliferative disease: A three-case series

**DOI:** 10.1016/j.rmcr.2025.102353

**Published:** 2025-12-26

**Authors:** Sanya Chandna, Rutvik Raval, Tanya Rangwala, Atul C. Mehta

**Affiliations:** a Department of Hospital Medicine, Cleveland Clinic Foundation, OH, USA; b Department of Internal Medicine, B.J. Medical College, Ahmedabad, India; c Respiratory Institute, Cleveland Clinic, Cleveland, OH, USA

**Keywords:** Nodular amyloidosis, Lymphoproliferative diseases

## Abstract

Pulmonary amyloidosis presents in nodular, tracheobronchial, and diffuse alveolar-septal forms. Nodular pulmonary amyloidosis (NPA) is usually localized and asymptomatic. It is often linked to indolent B-cell lymphoproliferative disorders (LPD). The National Comprehensive Cancer Network recommends long-term follow-up and evaluation for low-grade B-cell lymphoproliferative disorders when specific clinical features are present. However, there are no established screening guidelines specifically for detecting lymphoma in patients with nodular pulmonary amyloidosis. We describe three patients with NPA who later developed LPD. A 53-year-old woman with biopsy-proven κ-type localized AL amyloidosis was later found to have monoclonal gammopathy of undetermined significance. A 63-year-old man with prior hairy cell leukemia had λ-type NPA and subsequently Epstein–Barr virus–positive Hodgkin lymphoma. A 74-year-old man undergoing pneumothorax surgery had NPA with bronchus-associated lymphoid tissue lymphoma and later gastric mucosa-associated lymphoid tissue lymphoma. None of them had systemic amyloidosis at diagnosis. These cases suggest NPA may coexist with or precede LPD, underscoring the importance of longitudinal monitoring. Larger studies are needed to help standardize screening, as early identification of an underlying LPD could potentially improve hematologic outcomes.

## Highlights

• Nodular pulmonary amyloidosis is often incidental and may precede indolent B-cell lymphoproliferative disorders.
• Accurate diagnosis may require surgical biopsy due to amyloid deposits masking underlying lymphoid proliferation.
• Our case series emphasizes the need for longitudinal follow-up and structured surveillance for early hematologic diagnosis.

## Abbreviations

NPA: Nodular Pulmonary Amyloidosis
LPD: Lymphoproliferative Disorders
CT: Computed Tomography
MGUS: Monoclonal Gammopathy of Undetermined Significance
VATS: Video-Assisted Thoracic Surgery
EBV: Epstein–Barr Virus
CPAP: Continuous Positive Airway Pressure
EKG: Electrocardiogram
BALT: Bronchus-Associated Lymphoid Tissue
NSAIDs: Nonsteroidal Anti-Inflammatory Drugs
MALT: Mucosa-Associated Lymphoid Tissue

## 1 Introduction

Amyloidosis is a heterogeneous group of diseases marked by the accumulation of misfolded proteins extracellularly. Amyloid is an insoluble protein that primarily consists of a β-sheet structure. It possesses a distinctive affinity for the Congo Red dye and exhibits an apple-green birefringence when viewed under polarized light microscopy. It is capable of affecting the body systemically, or it may be confined to a particular area. Localized amyloidosis commonly impacts the larynx, trachea, lung, skin, and urinary tract. In lung-related cases, three types exist - nodular, tracheobronchial, and diffuse alveolar-septal. The first two are typically associated with localized pulmonary amyloidosis [1,2]. Nodular pulmonary amyloidosis (NPA), more prevalent than tracheobronchial amyloidosis, is frequently misdiagnosed as a tumor. Many experts suggest that most instances of NPA stem from an underlying lymphoproliferative disorder (LPD) [3]. However, no studies have conclusively established nodular amyloidosis as a precursor to lymphoproliferative disorders.

We present a case series involving three patients diagnosed with NPA, subsequently developing LPD.

## 2 Case presentation

### 2.1 Case 1

**Fig. 1:**
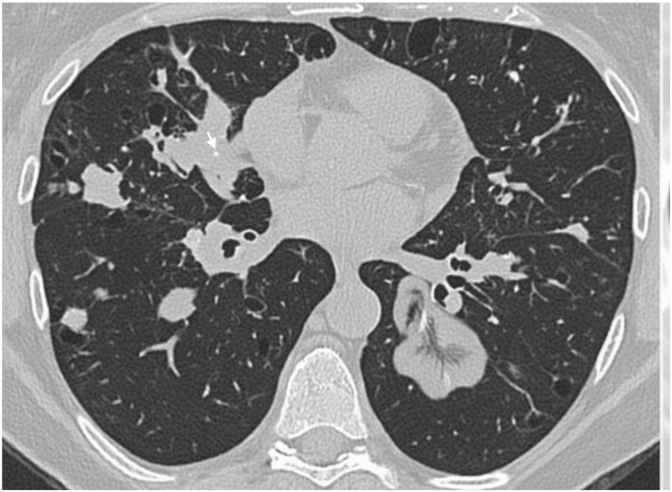
CT Chest shows multiple bilateral lung nodules, some with central calcifications (arrow).

A 53-year-old Caucasian female with no significant medical history presented to the pulmonary clinic with a 4-month history of dyspnea on exertion and dry cough. She was a lifelong non-smoker and worked as a medical transcriptionist. CT chest revealed multiple bilateral lung nodules (Fig. 1). She was started on prednisone for presumed sarcoidosis. After three months, she reported subjective improvement. However, a repeat chest CT showed the persistence of bilateral lung nodules. She ultimately underwent a CT-guided core needle biopsy of a right upper lobe lung nodule. The pathological examination demonstrated amyloid deposition. Amyloid typing identified κ light chain as the amyloidogenic protein. Prednisone was tapered off. She also underwent extensive lab testing. Her complete blood count, kidney function, and calcium level were within normal limits. Serum-free light chain assay was normal. Serum protein electrophoresis was positive for M-protein (0.98 mg/dL). Serum immunofixation revealed mildly elevated IgG [1620 mg/dL (ref. 717–1411 mg/dL)] and κ level [1290 mg/dL (ref. 534–1267 mg/dL)]. A 24-h urine protein was normal, and no M-protein was identified on urine protein electrophoresis. Bone marrow biopsy showed no evidence of clonal plasma cells. Given a normal serum light chain assay, normal bone marrow biopsy, and lack of systemic involvement, she was diagnosed with localized AL amyloidosis involving the lung. She was also diagnosed with monoclonal gammopathy of undetermined significance (MGUS) based on positive serum M-protein. No treatment was initiated, and she continued follow-up with pulmonology and oncology as an outpatient.

### 2.2 Case 2

A 63-year-old Caucasian male with a significant history of hairy cell leukemia, treated with chemotherapy status post splenectomy, in remission, presented with bilateral enlarging lung nodules found on a routine chest x-ray. He was completely asymptomatic. He smoked 3–4 cigarettes per week for 10 years and worked as a skier. He mentioned that roughly around 10 years ago, he was found to have lung nodules on chest imaging done at an outside hospital. He underwent a VATS lung biopsy 4 years ago at the same hospital, which showed nodular amyloid degeneration. Due to enlarging lung nodules, he was concerned if he had developed lung malignancy and hence came to our clinic. The chest x-ray revealed enlarging nodules in the right and left lung, with the dominant nodule in the right middle lobe measuring 3.8 × 4.3 cm and a retrocardiac nodule is 2.8 cm (Fig. 2a, Fig. 2ba & b). He underwent a transbronchial biopsy of the right middle lobe nodule, consistent with nodular amyloidosis. Amyloid typing identified λ light chain as the amyloidogenic protein. A normal bone marrow biopsy confirmed the absence of systemic amyloidosis involvement, leading to a diagnosis of AL amyloidosis. He continued to follow up at the clinic and get surveillance imaging. A subsequent CT scan of the abdomen done after a few years revealed a notable increase in retroperitoneal lymphadenopathy. Consequently, a diagnostic laparoscopy was performed, during which an excisional biopsy of the retroperitoneal lymph node was conducted. The biopsy results indicated EBV-positive Hodgkin lymphoma, specifically the nodular sclerosis type, with focal involvement by hairy cell leukemia. Notably, the congo red stain tested negative for amyloid. Subsequently, he commenced chemotherapy treatment comprising brentuximab-vedotin, adriamycin, vinblastine, and dacarbazine. He is currently undergoing regular follow-ups at the clinic.

**Fig. 2a:**
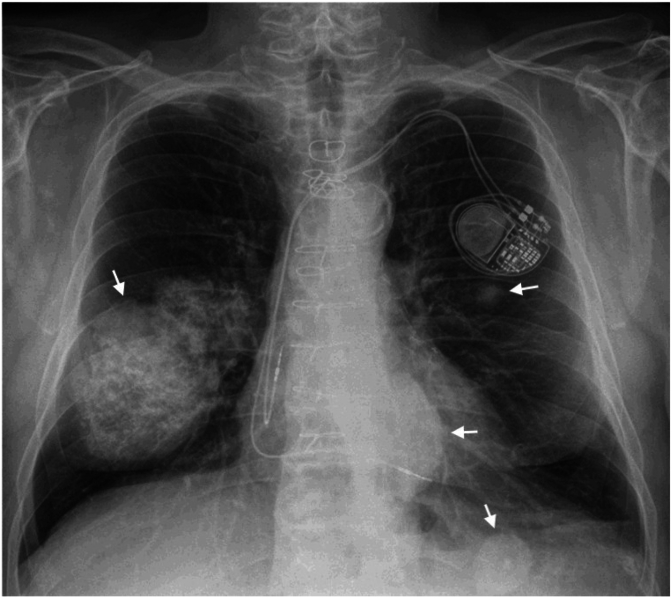
Chest radiograph revealing multiple calcified bilateral pulmonary nodules (arrows) of varying size.

**Fig. 2b:**
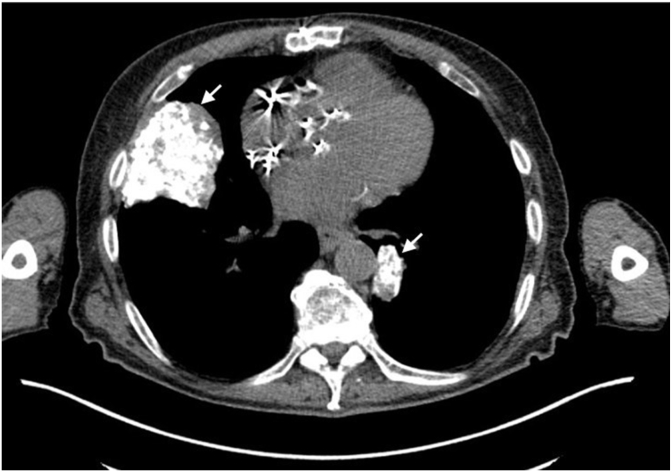
CT Chest confirming calcification in pulmonary nodules (arrows).

### 2.3 Case 3

**Fig. 3:**
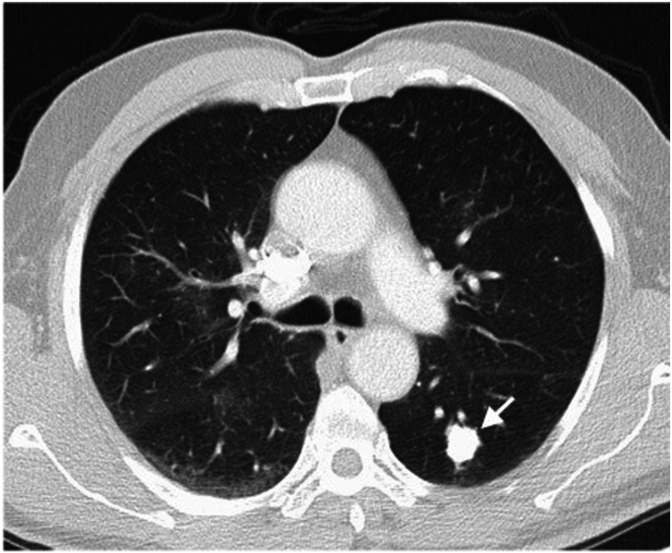
CT Chest revealing calcified left lower lobe nodule (arrow).

A 74-year-old Asian male with a medical history significant for bilateral calcified lung nodules, legionnaire's disease requiring prolonged hospitalization, obstructive sleep apnea on CPAP, hypertension, and hyperlipidemia presented to the emergency room with pleuritic chest pain and shortness of breath, which started a couple of hours prior. Vitals and lab work, including a complete blood count, comprehensive metabolic panel, troponin, and D dimer, were all normal. The EKG did not show any signs of acute ischemia. However, a chest x-ray revealed a significant left pneumothorax and left lung collapse. An 8 fr left anterior chest tube was inserted, but due to persistent pneumothorax, cardiothoracic surgery was consulted. A subsequent CT chest scan showed a large, loculated pneumothorax. The CT scan also shows calcified lung nodule (Fig. 3). The patient underwent VATS with left upper lobe bullectomy, left upper and lower lobe wedge resection, and left talc pleurodesis. The patient was already undergoing regular outpatient monitoring with a pulmonologist for the surveillance of his calcified lung nodules. Notably, he worked as an accountant and had no history of smoking. An infectious workup for the lung nodules done as an outpatient revealed negative results. While undergoing VATS, a biopsy of the lung nodule was performed as well. Pathology results showed extranodal marginal zone lymphoma of bronchus-associated lymphoid tissue (BALT lymphoma) and nodular amyloidosis with ossification. There were no signs of systemic amyloidosis. The patient continued regular follow-ups for surveillance imaging, which consistently showed stable CT chest findings. Additionally, he also followed up with an oncologist who did not recommend any specific treatment. Three years later, the patient experienced an upper gastrointestinal bleed due to the use of nonsteroidal anti-inflammatory drugs and steroids. An esophagogastroduodenoscopy revealed mucosa-associated lymphoid tissue (MALT lymphoma). Immunostaining was negative for *Helicobacter pylori*. Oncology team suggested no immediate treatment and opted for continued surveillance. The patient continues to follow up with both oncology and pulmonology for ongoing monitoring.

## 3 Discussion

NPA is form of localized pulmonary amyloidosis. Most patients are asymptomatic, and the condition is often found incidentally on imaging. When symptoms do occur, they may include cough, wheezing, shortness of breath, or recurrent infections [1]. Reports suggest that about 30–50 % of NPA cases are linked to an underlying LPD, most commonly low-grade B-cell lymphomas such as MALT lymphoma [[3], [4], [5], [6]]. In some cases, lung nodules may be the first sign of the disease [6,7]. The amyloid in these patients forms because a small group of abnormal B cells produces excess monoclonal light chains, sometimes heavy chains. These misfold and deposit in the lung as AL-type amyloid [3,4]. Distinguishing nodular pulmonary amyloidosis from lymphoma can be difficult because the heavy amyloid deposits often hide the underlying lymphoid process. Accurate diagnosis usually requires combining morphology, immunostains, and molecular studies. A surgical biopsy is often needed instead of a transbronchial biopsy, since larger tissue samples are usually required to detect a lymphoproliferative disorder that may be hidden by the amyloid deposits [8,9]. Localized AL amyloidosis generally has a favorable outcome and rarely progresses to systemic disease [10]. Treatment options include surgical removal, bronchoscopic debulking, and laser-based therapies [1]. Even in cases associated with lymphoma, the overall prognosis is usually favorable, although long-term monitoring is needed. The current literature shows that NPA is often associated with LPD, but no studies have definitively shown that NPA itself acts as a precursor to lymphoma.

Our first patient with lung nodules was initially misdiagnosed with sarcoidosis. A biopsy confirmed the diagnosis of NPA, and prednisone was tapered off. However, she was later diagnosed with MGUS. The second patient had a previous history of hairy cell leukemia, which was in remission. During routine screening, lung nodules were discovered. He was entirely asymptomatic. A biopsy confirmed the diagnosis of NPA. Given the lack of symptoms, monitoring was the chosen approach. Subsequently, after a few years, this patient was diagnosed with Hodgkin's lymphoma. The third patient, who was being monitored for lung nodules, underwent a biopsy solely due to developing a pneumothorax that required a VATS procedure. Incidentally, this procedure led to the diagnosis of both NPA and Bronchus Associated Lymphoid Tissue Lymphoma (BALToma). A few years later, this patient experienced a gastrointestinal bleed due to non-steroidal anti-inflammatory drugs and steroids and was incidentally diagnosed with gastric Mucosa-associated lymphoid tissue lymphoma (MALToma).

Although evidence is limited, our series raises the question of whether NPA may represent a precursor of a clonal B-cell process that becomes clinically apparent as lymphoma over time. It is possible that the lymphoma was present from the start, but was too small or subtle to detect at the time NPA was diagnosed. Chronic antigenic stimulation from infections, autoimmune disease, or ongoing inflammation may promote this clonal B-cell proliferation over time. One prior study also described a patient who developed MALT lymphoma eight years after excision of a cutaneous AL amyloidoma, supporting the idea that progression can occur [11].

Currently, there are no guidelines recommending routine screening for LPD in patients with NPA. Given the known association between localized AL amyloidomas and B-cell neoplasms, future recommendations could include periodic surveillance to detect hematologic malignancies earlier. In developing such recommendations, several factors should be considered. Risk stratification could include the presence of monoclonal proteins, clonal B-cell populations on biopsy, multiple or progressive nodules, or clinical symptoms suggestive of lymphoma. Follow-up should incorporate periodic clinical evaluation, laboratory studies such as serum and urine protein electrophoresis, immunofixation, and serum free light-chain assays, as well as imaging to monitor nodule progression. Adequate tissue sampling and careful pathological review are essential to detect subtle lymphoproliferative processes that may be obscured by amyloid deposits. Finally, patient education regarding symptoms that should prompt evaluation and clearly defined follow-up intervals is an important component of any surveillance strategy. Larger studies are needed to establish evidence-based guidelines for early detection and intervention in this patient population.

## 4 Conclusion

There are no guideline-directed surveillance intervals for screening patients with localized amyloidoma for lymphoproliferative disorders. Although progression to systemic amyloidosis is uncommon, the potential development of an underlying lymphoproliferative disorder warrants long-term follow-up. Ongoing monitoring should include periodic clinical evaluation, serial testing for monoclonal proteins, assessment of organ function, and imaging when clinically indicated.

## CRediT authorship contribution statement

**Sanya Chandna:** Writing – review & editing, Writing – original draft, Conceptualization. **Rutvik Raval:** Writing – review & editing, Writing – original draft. **Tanya Rangwala:** Writing – original draft. **Atul C. Mehta:** Writing – review & editing, Supervision, Data curation, Conceptualization.

## Ethical approval

None.

## Consent

Written consent is available upon request.

## Guarantor

Dr. Atul C. Mehta (Corresponding Author).

## Funding

None.

## Declaration of competing interest

The authors declare that they have no known competing financial interests or personal relationships that could have appeared to influence the work reported in this paper.

## References

[1] Patel H., Sheikh A., Medarametla G.D. (2023). Uncommon presentation of undiagnosed B-Cell lymphoproliferative disorder as nodular pulmonary amyloidosis. J. Med. Cases.

[2] Ding F., Li Y., Balasubramanian S. (2021). A unique case of combined nodular and tracheobronchial amyloidosis. Oxford Med Case Reports.

[3] Grogg K.L., Aubry M.C., Vrana J.A., Theis J.D., Dogan A. (2013). Nodular pulmonary amyloidosis is characterized by localized immunoglobulin deposition and is frequently associated with an indolent B-cell lymphoproliferative disorder. Am. J. Surg. Pathol..

[4] Ryan R.J., Sloan J.M., Collins A.B., Mansouri J., Raje N.S., Zukerberg L.R., Ferry J.A. (2012 Jan). Extranodal marginal zone lymphoma of mucosa-associated lymphoid tissue with amyloid deposition: a clinicopathologic case series. Am. J. Clin. Pathol..

[5] Lim J.K., Lacy M.Q., Kurtin P.J., Kyle R.A., Gertz M.A. (2001 Aug). Pulmonary marginal zone lymphoma of MALT type as a cause of localised pulmonary amyloidosis. J. Clin. Pathol..

[6] Dacic S., Colby T.V., Yousem S.A. (2000 Sep). Nodular amyloidoma and primary pulmonary lymphoma with amyloid production: a differential diagnostic problem. Mod. Pathol..

[7] Upadhaya S., Baig M., Towfiq B., Al Hadidi S. (2017).

[8] Filippi N., Diotti C., Donghi S.M., Galetta D., Sedda G., Sandri A., De Camilli E., Guarize J., Spaggiari L. (2019 May). Diagnostic and therapeutic implications of pulmonary lymphoma associated with nodular amyloidosis. Ann. Thorac. Surg..

[9] Lantuejoul S., Moulai N., Quetant S., Brichon P.Y., Brambilla C., Brambilla E., Ferretti G.R. (2007 Sep). Unusual cystic presentation of pulmonary nodular amyloidosis associated with MALT-type lymphoma. Eur. Respir. J..

[10] Milani P, Basset M, Russo F, Foli A, Palladini G, Merlini G. The Lung in Amyloidosis. doi:10.1183/16000617.0046-2017. 10.1183/16000617.0046-2017 PMC9488920 28877975

[11] Walsh N.M., Lano I.M., Green P., Gallant C., Pasternak S., Ly T.Y., Requena L., Kutzner H., Chott A., Cerroni L. (2017 Aug). AL Amyloidoma of the Skin/Subcutis: cutaneous amyloidosis, plasma cell Dyscrasia or a manifestation of primary cutaneous Marginal Zone lymphoma?. Am. J. Surg. Pathol..

